# 610. Retrospective Safety Evaluation of Intramuscular Testosterone Cypionate in Male Burn Patients

**DOI:** 10.1093/jbcr/irag033.421

**Published:** 2026-04-06

**Authors:** Melissa A Reger

**Affiliations:** Leon S. Peters Burn Center, Fresno, CA

## Abstract

**Introduction:**

Severe burn injury triggers a hypermetabolic state characterized by profound catabolism, muscle wasting, and impaired wound healing. Following the 2023 market withdrawal of oxandrolone, our burn center implemented intramuscular (IM) testosterone cypionate as an alternative anabolic agent. We aimed to evaluate its safety in male burn patients by assessing metabolic, hepatic, hematologic, and cardiovascular outcomes.

**Methods:**

This single-center, retrospective review included adult male burn patients who received at least two doses of IM testosterone cypionate 200 mg and had a documented baseline (pre-treatment) and at least one follow-up total testosterone level. Outcomes included changes in testosterone levels, liver enzymes, hemoglobin, blood pressure, and the incidence of adverse events such as thrombotic complications, injection site reactions, or glycemic disturbances.

**Results:**

Five patients met inclusion criteria. Median age was 40 years and median total body surface area burn was 30%. All patients were hypogonadal at baseline, with improvement in testosterone levels after at least two doses. Mild transaminitis occurred in all patients, possibly due to concurrent hepatotoxic medications. No significant hematologic, thrombotic, or cardiovascular complications were identified. None of the patients experienced an injection site reaction or developed hyperglycemia.

**Conclusions:**

IM testosterone cypionate appears to be a safe and feasible alternative anabolic strategy for male burn patients when carefully monitored. The expected rise in testosterone was achieved without major safety concerns, though mild transaminitis was common and likely multifactorial. While these findings are reassuring from a safety perspective, the clinical efficacy of testosterone compared with oxandrolone remains unknown. Larger studies are needed to confirm safety, establish efficacy, and define optimal dosing and monitoring in this population.

**Applicability of Research to Practice:**

The withdrawal of oxandrolone left burn centers with limited anabolic options to address the hypermetabolic response in severe burn injury. This study demonstrates that intramuscular testosterone cypionate can be a feasible and safe alternative when used with appropriate monitoring. However, its clinical efficacy compared to oxandrolone remains unknown, and larger studies are needed to determine whether testosterone provides similar benefits in wound healing, lean mass preservation, and length of stay.

**Funding for the study:**

N/A.

